# Exercise training and cardiac autonomic function following coronary artery bypass grafting: a systematic review and meta-analysis

**DOI:** 10.1186/s43044-022-00306-5

**Published:** 2022-09-23

**Authors:** Purnima Kushwaha, Jamal Ali Moiz, Aqsa Mujaddadi

**Affiliations:** Centre for Physiotherapy and Rehabilitation Sciences, Jamia Millia Islamia (A Central University), New Delhi, 110025 India

**Keywords:** Autonomic nervous system, Exercise training, Heart rate variability, Heart rate recovery, Coronary artery bypass graft

## Abstract

**Background:**

Exercise training improves cardiac autonomic function is still debatable in patients with coronary artery bypass grafting (CABG). The aim of the present review is to assess the effect of exercise on CABG patient’s heart rate variability (HRV) and heart rate recovery (HRR) parameters.

**Main body:**

Databases (PubMed, Web of Science and PEDro) were accessed for systematic search from inception till May 2022. Eleven potential studies were qualitatively analyzed by using PEDro and eight studies were included in the quantitative synthesis. Meta-analysis was conducted by using a random-effect model, inverse-variance approach through which standardized mean differences (SMDs) were estimated. The analysis of pooled data showed that exercise training improved HRV indices of standard deviation of the R–R intervals (SDNN) [SMD 0.44, 95% CI 0.17, 0.71, *p* = 0.002], square root of the mean squared differences between adjacent R–R intervals (RMSSD) [SMD 0.68, 95% CI 0.28, 1.08, *p* = 0.0008], high frequency (HF) [SMD 0.58, 95% CI 0.18, 0.98, *p* = 0.005] and low frequency-to-high frequency (LF/HF) ratio [SMD − 0.34, 95% CI − 0.65, − 0.02, *p* = 0.03].

**Conclusions:**

Exercise training enhances cardiac autonomic function in CABG patients. Owing to the methodological inconsistencies in assessing HRV, the precise effect on autonomic function still remains conflicted. Future high-quality trials are needed focusing on precise methodological approach and incorporation of various types of exercise training interventions will give clarity regarding autonomic adaptations post-exercise training in CABG.

*Trial registration* CRD42021230270, February 19, 2021.

## Background

Cardiovascular diseases (CVDs) are the leading cause of death around the world. The World Health Organization (WHO) said that CVDs were the main cause of about 17.9 million deaths (32%) in 2019 [1]. Coronary artery bypass grafting (CABG) is a successful procedure for declining the manifestations and mortality of coronary artery disease (CAD) [2]. Alteration in cardiac autonomic control occurs due to CABG procedures such as cardiopulmonary bypass, thoracic contents manipulation [3], sustained anesthesia, cardioplegia, extracorporeal circulation and bed rest following surgery [4]. Increased sympathetic drive and depressed vagal activity ultimately result in adverse outcomes [5]. Therapies that enhance autonomic balance including pharmacological or exercise interventions are therefore of interest in the management of CABG.

Several assessment approaches such as heart rate variability (HRV) and heart rate recovery (HRR) can be used to determine cardiac autonomic function. HRV is the term used to describe the variation in the time between subsequent heartbeats (R–R interval) [6]. In post-CABG patients, significant impairment in cardiac autonomic control was observed by HRV [4, 7–9]. Lower HRV indicates an intrinsic impairment with the heart’s sinoatrial node’s rhythm control, making the subject less tolerant of physiological homeostasis including an ischemic episode or more regular cardiac electrophysiology disturbances [10]. Following CABG, HRV indices may be reduced for months or years prior returning to preoperative levels, as indicated by previous trials [4, 11, 12]. An imbalance in HRV increases the risk of hemodynamic dysfunction, arrhythmias and sudden death [13]. HRR stands for heart rate reduction rate which is decreased after exercise [14]. A greater death rate is independently predicted by an abnormal HRR which is connected to diminished vagal activity [15]. As a result, in these patients treatments that contribute to the improved cardiac autonomic activity as fast as possible following surgery may be clinically significant.

The electrocardiogram (ECG) R–R interval variation and overall spontaneous baroreflex are autonomic markers of sinoatrial node neural regulation that are significantly improved by exercise-based cardiac rehabilitation [16–19]. Exercise has been proven to alter the sympathovagal control of heart rate hence improving parasympathetic tone. Additionally, reduced cardiac event mortality is linked to improved vagal activity [20, 21]. Even though exercise training is advised as an adjuvant to medical therapy after CABG, there is little published research to evaluate cardiac autonomic function after exercise training in CABG. Current exercise therapies may be seen as an appealing and advantageous option for the elimination and management of autonomic abnormalities in patients after CABG due to their low cost and easy accessibility. Hence, the purpose of this systematic review and meta-analysis is to explore how exercise training affects cardiac autonomic function in post-CABG patients.

Preferred Reporting Items for Systematic Reviews and Meta-Analyses lists the design and methodology (PRISMA). The PICO criteria were used to create the eligibility assessment. The current protocol for a systematic review and meta-analysis has been registered on the PROSPERO database (CRD42021230270). Searching was conducted on the electronic databases such as PubMed, Web of Science and PEDro from the inception to May 22, 2022 using different combinations of keywords including “Coronary artery bypass graft” OR “bypass surgery” AND “exercise training” OR “resistance” OR “aerobic” OR “strength” OR “endurance training” OR “interval training” OR “physical training” AND “heart rate variability” OR “heart rate recovery” OR “autonomic function” OR “sympathetic function”. On PEDro database, we performed simple search strategy using different terms “Coronary artery bypass grafting *exercise * autonomic function”. We limited the search results to RCT. To find further full-text articles we screened the reference lists of the original and review studies.

We formulated eligibility criteria using the PICOS (populaton, intervention, comparisons, outcomes and study design) guidelines. We looked at the effects of different types of exercise training including aerobic, resistance, interval, and combined aerobic and resistance training either alone or in conjunction with psychosocial and/or educational interventions on adult CABG patients (population) which included both male and female patients (> 18 years). We did not set any restrictions on how long exercise-based cardiac rehabilitation (CR) should last while analyzing the efficacy of rehabilitation on autonomic function (intervention). Routine medical treatments, psychosocial therapies, and/or educational programs may be part of the comparison group but there will be no formal exercise training program (comparison). The HRV, HRR or both have to be reported in each included study. For HRV, we selected those studies which reported three time domain variables, namely standard deviation of the R–R intervals (SDNN), standard deviation of the 5 min mean R–R intervals (SDANN) and square root of the mean squared differences between subsequent R–R intervals (RMSSD) that measure overall HRV, sympathetic activity and vagal activity along with three frequency domain variables, namely low frequency (LF), high frequency (HF) and LF/HF ratio measuring a combination of sympathetic and parasympathetic input, vagal function and the global sympathovagal balance (< 1), respectively. The studies were relevant if they examined HRR for the first minute following maximal activity (outcomes). We included only randomized controlled trials (study design). Trials that were non-randomized, had no control group, review articles or case studies, epidemiological studies (cross-sectional and cohort), were written in a language other than English and included breathing exercises, yoga, Tai Chi and different kinds of physical therapy were excluded.

Retrieved articles from each database were imported to the Mendeley software where the articles were compiled and duplicates were eliminated. As per the inclusion and exclusion criteria, two authors (P.K. and A.M.) identified the study titles and abstracts that were chosen in an electronic search. After abstracts were reviewed, the same authors got the full texts of eligible articles by separately reapplying inclusion and exclusion criteria. The author P.K. went through the references of the full-text papers chosen for the review to get a final list of publications to use in the review. Consensus was reached with a third reviewer to settle disagreements (J.M.).

Data was extracted which included the details on study population (CABG), demographic data (age, gender), details of the intervention (type of exercise, frequency, intensity, length of sessions in minutes, length of treatment in weeks, setting if supervised hospital/center- or home-based, and wait time to start exercise-based CR after operation or event would be < 3 months or ≥ 3 months), details of the control group (usual care or “simulated” care, such as breathing exercises), primary outcome measures (HRV, HRR), and the main findings were retrieved from the study independently by two authors (P.K. and A.M.).

RevMan 5.4 software was used to carry out the quantitative analysis. Only studies with sufficient data on any of the predetermined outcome measures were included in the meta-analysis. The effect size estimates for HRV and HRR were calculated using the standardized mean difference (SMD) [SMD = MD/SD pooled], where MD is the difference in means between the intervention and control groups and SD is the pooled standard deviation. The random-effects inverse variance method was used since it is a more conventional approach that considers the possibility that study heterogeneity can vary more than by chance. Only the final data for the intervention and control groups were retrieved if data were collected more than once during the intervention. Effect size was estimated using Cohen’s d criterion (small, 0.2; moderate, 0.2–0.5; or large, > 0.8) [22]. The *I*^2^ test was used to evaluate the degree of study heterogeneity and to ascertain the percentage of observed variability across effect estimates that is not random. An *I*^2^ value of less than 25%, between 25 and 75% and greater than 75% denote low, moderate and high heterogeneity respectively [23].

The authors used an 11-point PEDro scale designed to grade the quality of RCTs on the Physiotherapy Evidence Database to determine the methodological quality of the evidence. The effectiveness of the trials was graded independently by two reviewers. Each reviewer separately re-evaluated a criterion if there was a disagreement regarding it. Each criterion was scored either yes (score = 1) or no (score = 0) to reduce ambiguity in responses. The total responses were used to calculate the methodological quality score for each included study (maximum score 10). Studies were rated as having poor quality (score of 4), fair quality (score of 4–5), good quality (score of 6–8) or excellent quality (score of > 8) [24].

## Main text

**Fig. 1:**
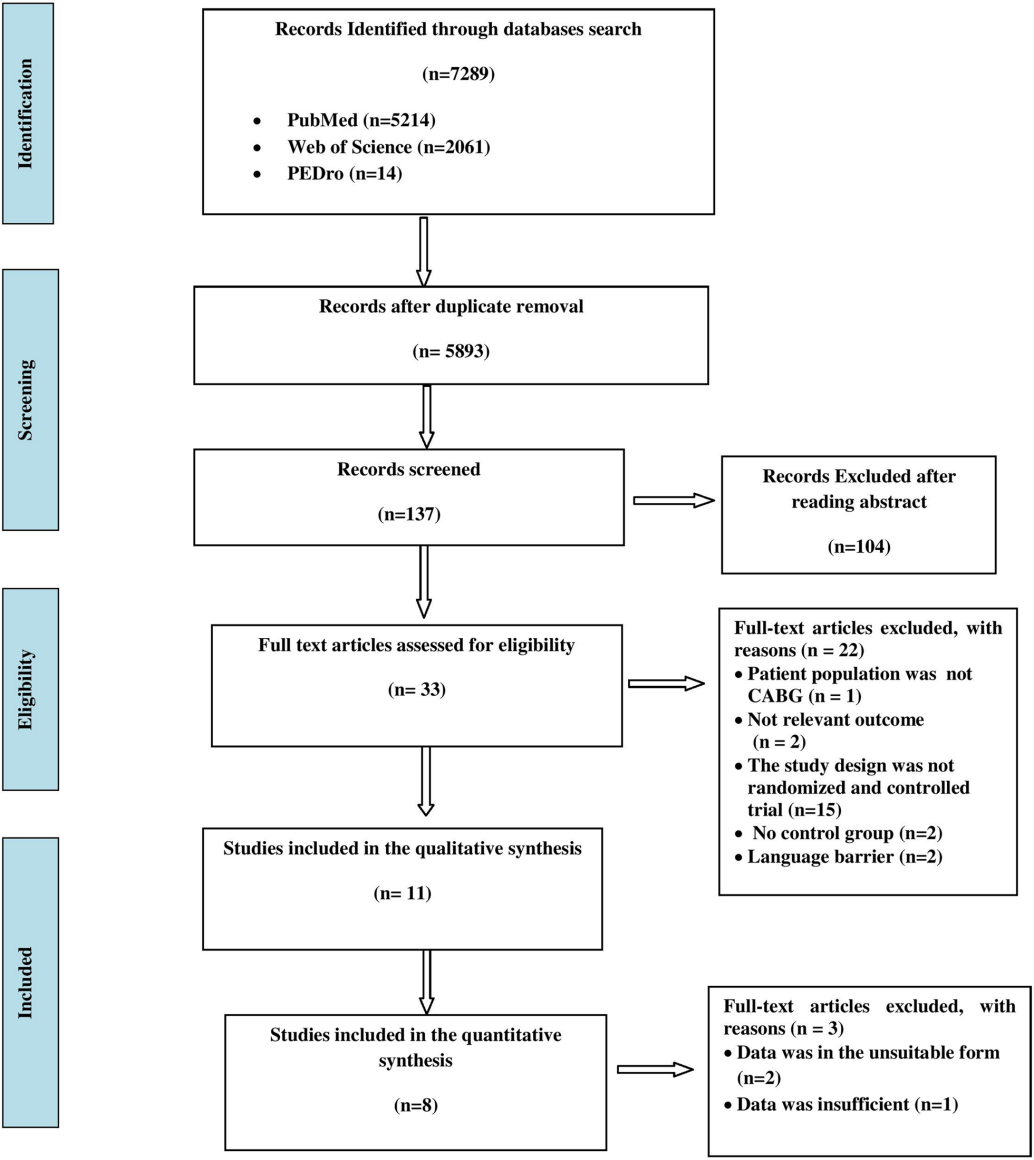
PRISMA flowchart

The initial search yielded 7289 results, and after duplicates removal, 5893 were filtered by title and 137 articles were chosen. Thirty-three prospective studies were incorporated in the full-text analysis after the abstracts of the chosen results were reviewed. Twenty-two studies were not included because they lacked a population sample [25], assessed outcomes not pertinent to the review [26, 27], lacked a randomized and controlled design [28–42], lacked a control group [43, 44] and were not in English [45, 46]. For the qualitative synthesis, eleven studies were used [47–57] out of which only eight studies [47–49, 51–54, 56] were included for meta-analysis as data provided in the remaining three studies were in unsuitable form [50, 55] and insufficient [57]. The PRISMA 2009 flow diagram depicts the study selection procedure (Fig. 1).

The eleven studies included were randomized controlled trials. Each study included a control group and an exercise group. Routine medical treatment, breathing exercises, or no exercise at all were given to the control group. In the systematic review, the details of the studies included are described (Table 1).

**Table 1:** Overview of the randomized controlled studies that were included (*n* = 11)

Study	Participants	Intervention	Outcome measures	Main findings
Wu et al. [48]	After > 1 week CABG, *n* = 54 randomized and analyzed E1: *n* = 18 (100% male) 62.8 ± 6.9 years E2: *n* = 18 (100% male) 60.9 ± 7.6 years C: *n* = 18 (100% male) 62.2 ± 9.6 years 30% β-blocker (E1: *n* = 5, E2: *n* = 7, C: *n* = 4)	12 weeks, aerobic exercise 3 sessions per week E1: (CR) 30 to 60 min at 60 to 85% of peak heart rate E2: (HBE) 30 to 60 min at 60 to 85% of peak heart rate C: continued with ADL	HRR_1_	↑ HRR_1_ in E1 and E2 groups
Tsai et al. [47]	After 1 week CABG, *n* = 30 randomized and analyzed E: *n* = 15 (NR) 61.23 ± 9.49 years C: *n* = 15 (NR) 63.23 ± 14.61 years No specific details on medications	E: 3 months aerobic exercise (stationary bicycle/walking on a treadmill) for 30 to 40 min, 3x/week at 60 to 85% of peak heart rate C: only phase 1 mobilization after surgery provided	HRR_1_	↑ HRR_1_ in E group
Legramante et al. [50]	After 1 week CABG, *n* = 82 randomized and analyzed E(TR): *n* = 43 (100% male) 59.6 ± 8.6 years C(UTR): *n* = 39 (100% male) 58.0 ± 7.5 years 14% β-blockers (E: *n* = 4, C: *n* = 8)	E: two daily sessions of 30 min of stationary cycling 6x/week for 2 weeks combined with calisthenics at 85% of the HR_max_ reached in the initial CPX (or to 75% of HR_max_ in patients older than 65 yrs) C: followed up the same daily calisthenics and walking routine schedule (2 daily sessions for 6x/week for 2 weeks) as the TR group	HRR_1_ HRR_2_	↔ HRR_1_ ↑ HRR_2_ in E group
Mehani [57]	After 4th day CABG, *n* = 50 randomized and analyzed E: *n* = 25 (100% male) 50.6 ± 8.6 years C: *n* = 25 (100% male) 50 ± 7.5 years 14% β-blockers (E: *n* = 4, C: *n* = 3)	E: ten steps cardiac rehabilitation program with adjusted intensity about 85% of maximal heart rate C: followed up the same daily mild calisthenics and walking routine, breathing exercises without adjustment for exercise intensity	HRR_1_	↑ HRR_1_ in E group
Bilińska et al. [51]	After 3 months CABG, *n* = 120 randomized, *n* = 100 analyzed E: *n* = 50 (100% male) 57 ± 6 years C: *n* = 50 (100% male) 56 ± 6 years 100% β-blocker	E: 6 weeks aerobic exercise(cycling) for 60 min, 3x/week at 70 to 80% of MHR C: Continued with ADL	HRV (SDNN, LF, HF, LF/HF) HRR_1_, HRR_2_	↑ SDNN, ↔ LF, ↔ HF, ↔ LF/HF in E group ↑ HRR_1_, ↑ HRR_2_ in E group (long-term recording)
Ghardashi-Afousi et al. [52]	After > 6 weeks CABG, *n* = 54 randomized, *n* = 42 analyzed E1(LV-HIIT): *n* = 14 (100% male) 53.90 ± 3.44 years E2(MICT): *n* = 14 (100% male) 54.10 ± 4.02 years C: *n* = 14 (100% male) 58.80 ± 4.41 years 28% β-blocker (E1: *n* = 3, E2: *n* = 4, C: *n* = 5)	E1 (LV-HIIT): 10 intervals of 2 min at 85 to 95% of HR_max_ and separated by 2 min at 50% HR_max_ for 3x/week for 6 weeks E2 (MICT): 40 min running on a treadmill at 70% of HR_max_ 3x/week for 6 weeks C: Continued with ADL	HRV (SDANN, RMSSD, LF, HF and LF/HF)	↑ SDANN, ↑RMSSD, ↑ HF, ↓ LF and ↓ LF/HF in E1 and E2 groups ↑ SDANN, ↑ HF, ↓ LF and ↓ LF/HF in E1 compared to E2 group (long-term recording)
Shao et al. [53]	After CABG, *n* = 53 randomized and analyzed E: *n* = 28 (64% male) 62.52 ± 7.05 years C: *n* = 25 60% male 64.47 ± 8.12 years No specific data provided on medications	E: 8 weeks, aerobic exercise (frequency and intensity-NR) C: Continued routine treatment for 8 weeks	HRV (SDNN, SDANN, RMSSD, PNN50)	↑SDNN, ↑SDANN ↑RMSSD, ↑PNN50 in E group (long-term recording)
Iellamo et al. [49]	After 1 week CABG, *n* = 97 randomized, *n* = 86 analyzed E(TR): *n* = 45 (100% male) 59.46 ± 7.8 years C(UTR): *n* = 41 (100% male) 58.56 ± 7.3 years 14% β-blocker (E: *n* = 4, C: *n* = 8)	E: Two daily sessions of 30 min of stationary cycling 6x/week for 2 weeks combined with calisthenics at 85% of the HR_max_ reached in the initial CPX (or to 75% of HR_max_ in patients older than 65 yrs) C: Continued to perform walking and calisthenics with the same daily schedule (2 daily sessions for 6x/week for 2 weeks as the TR group)	HRV (SDANN)	↑SDANN in E group (short-term recording)
Takeyama et al. [54]	After 1 week CABG, *n* = 28 randomized and analyzed E: *n* = 13 (100% male) 58.8 ± 6.3 years C: *n* = 15 (87% male) 61.7 ± 8.7 years 0% β-blocker	E: 30 min aerobic exercise (cycling) 2x/day at the anaerobic threshold for 2 weeks C: Walk 200 m 3x/day and progress to 500 m within 2 weeks	HRV (HF)	↑HF at rest after 3 months in E group ↑HF during exercise after 3 weeks in E group (short-term recording)
Mendes et al. [55]	After CABG, *n* = 74 randomized, *n* = 47 analyzed E: *n* = 24 (100% male) 60 ± 8 years C: *n* = 23 (100% male) 58 ± 9 years 74% β-blockers (E: *n* = 18, C: *n* = 17)	E: 5 days supervised inpatient physiotherapy protocol (active assisted, active exercises and ambulation gradually) C: deep breathing exercises	HRV (SDNN, SDANN RMSSD; LF, HF and LF/HF)	↑SDANN, ↑SDNN, ↑RMSSD, ↑HF, ↓LF, ↓LF/HF in the E group (short-term recording)
Ribeiro et al. [56]	After CABG, *n* = 70 randomized, *n* = 48 analyzed E1 (EMG): *n* = 15 (86% male) 58.3 ± 7.7 years E2 (VRG): *n* = 17 (59% male) 62.1 ± 9.0 years C: *n* = 16 (68% male) 60.3 ± 8.3 years 43% β-blockers (E1: *n* = 6, E2: *n* = 8, C: *n* = 7)	3 days of inpatient physiotherapy protocol E1: EMG (sitting out of bed, foot and ankle exercise, cycle ergometer, and ambulation) E2: VRG (similar protocols EMG with the addition of two Nintendo Wii games. A boxing game and the game “Basic Run” marching on the spot) C: Respiratory physiotherapy and foot and ankle exercises	HRV (SDNN, RMSSD; LF, HF, and LF/HF)	↑SDNN, ↑RMSSD, ↑HF,↓LF, ↓LF/HF ratio in E1 and E2 groups (short-term recording)

ADL, activities of daily living; C, control; CABG, coronary artery bypass graft; CPX, cardiopulmonary exercise testing; CR, cardiac rehabilitation; E, exercise; EMG, early mobilization group; HBE, home-based exercise; HF, high frequency; HR_max_, maximum heart rate; HRR_1_, heart rate recovery in the first minute after exercise; HRR_2_, heart rate recovery in the second minute after exercise; HRV, heart rate variability; LF, low frequency; LV-HIIT, low-volume high-intensity interval training; MICT, moderate-intensity continuous training; NR, not reported; pNN50, percentage of differences between adjacent NN intervals that are > 50 ms; RMSSD, root mean square of the difference in RR intervals; SDANN, standard deviation of the 5 min mean RR intervals; SDNN, standard deviation of all RR intervals; TR, trained; UTR, untrained; VRG, Virtual reality group; ↓, statistically significant decrease; ↑, statistically significant increase; and ↔, no statistically significant change

### Participants

In total, 620 participants from 11 studies—with sample population ranging from 28 to 100 and an average age of 50 to 64 years—were reported, with 339 in the exercise group and 281 in the control group. Eight trials had only male participants [48–52, 55–57] two trials [53, 54] had both sexes, and one trial [47] did not specify the gender of the participants. CABG surgery had been performed on all of the participants. The proportion of patients taking beta-adrenergic blocking drugs ranged from 0 to 100%, according to 9 trials (90.90%) [48–52, 54–57].

### Intervention

One study [52] compared low-volume high-intensity interval training (LV-HIIT) and moderate-intensity continuous training (MICT) as the aerobic exercise with the contro, and three studies [55–57] compared a short-term in-patient physiotherapy protocol with the control group. Of the 11 studies, 7 studies [47–51, 53, 54] compared aerobic exercise with the control group. A treadmill was employed in LV-HIIT. Exercise intensity for aerobic exercise and LV-HIIT and MICT is given in Table 1. The exercise intervention lasted between 3 days and 12 weeks, varied in length from 30 min to 60 min per day and occurred on average 3 to 7 days per week.

### Autonomic function assessment

Six studies evaluated and reported HRV [49, 52–56] as an evaluation of cardiac autonomic function and four studies assessed post-exercise HRR [47, 48, 50, 57], while one study assessed HRV in addition to HRR [51]. The method of assessment of HRV in three of the studies was 24 h Holter recording (long-term recording) [51–53] and four studies were short-term recording [49, 54–56]. Time domain was reported in six studies [49, 51–53, 55, 56] and frequency domain was reported in five studies [51, 52, 54–56]. The measurement of HRR_1_ was calculated after peak cardiopulmonary exercise testing or stress exercise testing by a cycle ergometer during a recovery phase in majority of the studies except one study [57] which measured HRR_1_ after six-minute walk test.

### Outcome measures

An outline of all pooled data analyses is given in Table 2.

**Table 2:** An outline of all pooled data analysis for HRV and HRR measures

ANS measures	Studies included	No. of participants		SMD (95% CI)
		Exercise	Control	
HRV				
Time domain				
SDNN	4	107	105	SMD 0.44 (0.17, 0.71), *p* = 0.002
SDANN	3	87	80	SMD 1.84 (− 0.11, 3.79), *p* = 0.07
RMSSD	3	57	55	SMD 0.68 (0.28, 1.08), *p* = 0.0008
Frequency domain				
LF	3	79	80	SMD − 1.50 (− 1.10, 0.10), *p* = 0.10
HF	4	92	95	SMD 0.58 (0.18, 0.98), *p* = 0.005
LF/HF	3	79	80	SMD − 0.34 (− 0.65, − 0.02), *p* = 0.03
HRR_1_	3	83	83	SMD 0.71 (0.39, 1.02), *p* < 0.0001

### Heart rate variability

#### Time domain parameter

##### SDNN

**Fig. 2:**
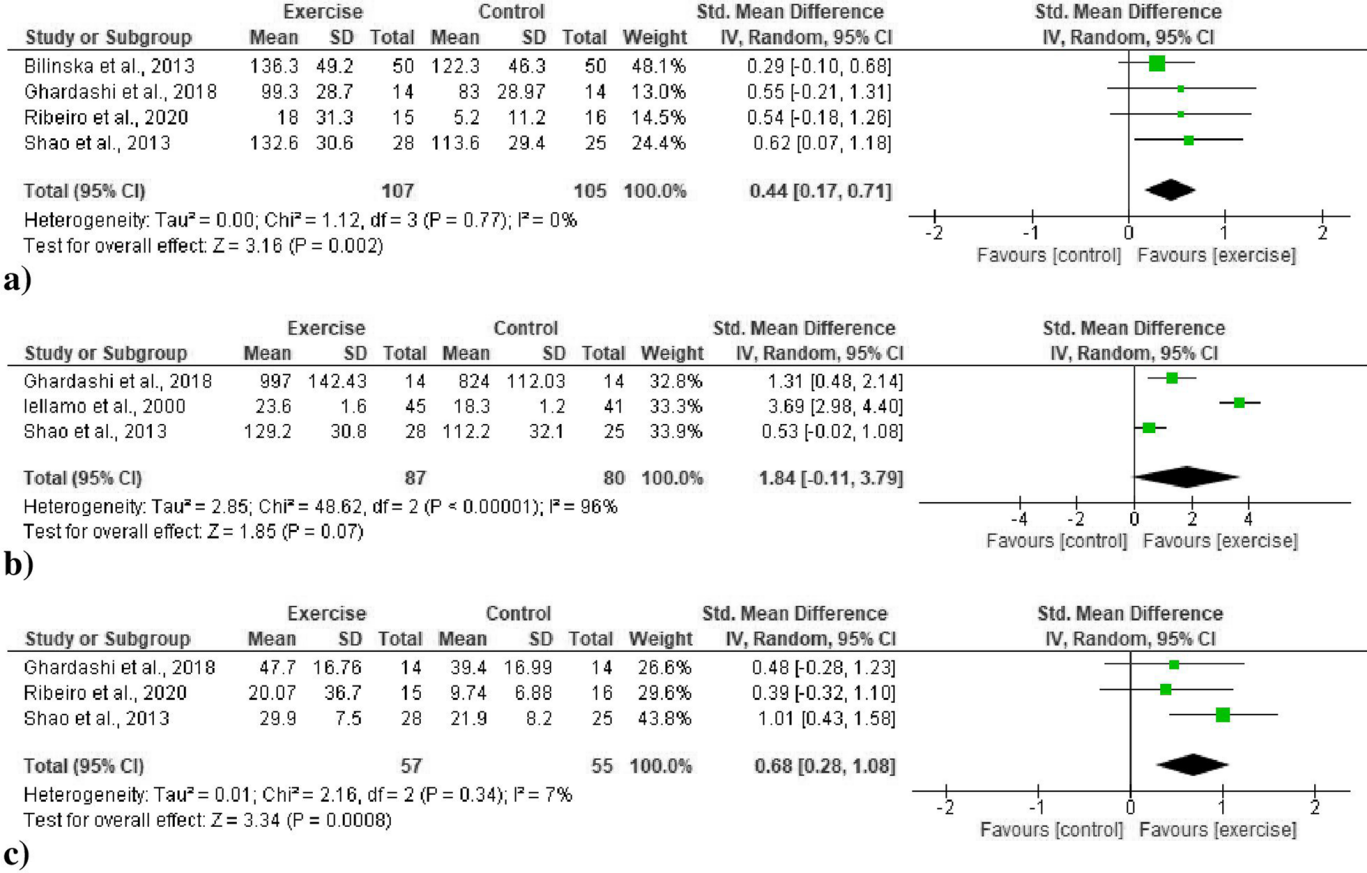
Forest plots for time domain HRV indices. **a** Overall changes in standardized mean difference indices for the standard deviation of all RR intervals (SDNN). **b** Overall changes in standardized mean difference indices for the standard deviation of the 5-min mean RR intervals (SDANN). **c** Overall changes in standardized mean difference indices for square root of the mean squared differences between successive RR intervals (RMSSD)

All data from four studies [51–53, 56] (107 exercise, 105 control participants) revealed a moderate significant improvement in SDNN in favor of the exercise training group (SMD 0.44, 95% CI 0.17, 0.71, *p* = 0.002) (Fig. 2a). The heterogeneity was statistically non-significant (*p* = 0.77), and the inconsistency was low (0%).

##### SDANN

All data from three studies [49, 52, 53] (87 exercise and 80 control participants) revealed a large but non-significant increase in the SDANN value (SMD 1.84, 95% CI − 0.11, 3.79, *p* = 0.07) (Fig. 2b). The heterogeneity was statistically significant (*p* < 0.00001), and the inconsistency was high (96%).

##### RMSSD

All data from three studies [52, 53, 56] (57 exercise, 55 control participants) revealed a moderate significant improvement in RMSSD in favor of the exercise training group (SMD 0.68, 95% CI 0.28, 1.08, *p* = 0.0008) (Fig. 2c). The heterogeneity was statistically non-significant (*p* = 0.34), and the inconsistency was low (7%).

### Frequency domain parameters

#### Low frequency (LF)

**Fig. 3:**
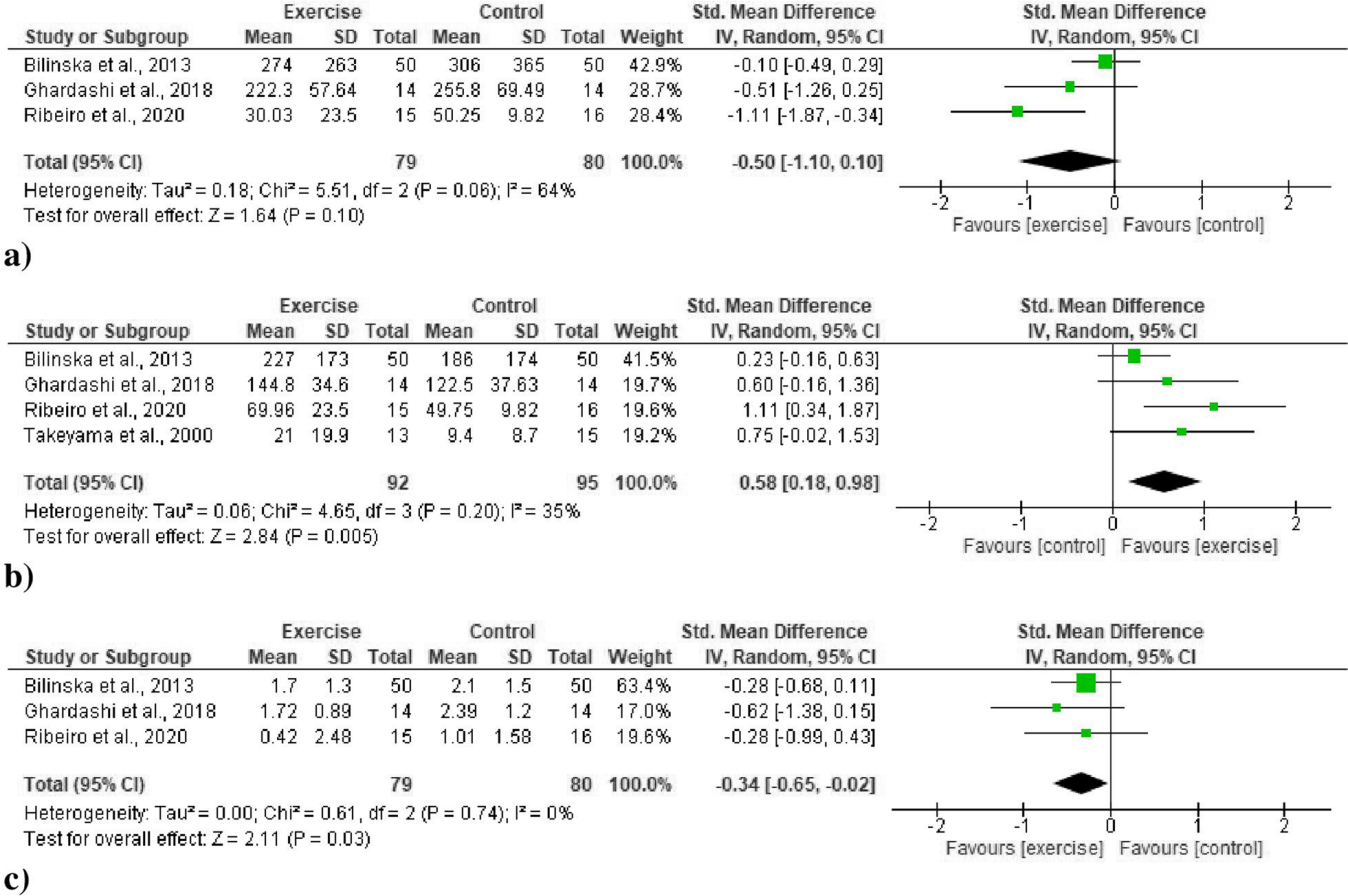
Forest plots for frequency domain HRV indices. -**a** Overall changes in standardized mean difference indices for low frequency (LF). **b** Overall changes in standardized mean difference indices for high frequency (HF). **c** Overall changes in standardized mean difference indices for LF/HF ratio

Pooled data from three studies [51, 52, 56] (79 exercise, 80 control participants) revealed an overall moderate decrease (improvement) in the LF value, but the result was statistically non-significant (SMD − 0.50, 95% CI − 1.10, 0.10, *p* = 0.10) (Fig. 3a). The heterogeneity was statistically non-significant (*p* = 0.06), and the inconsistency was moderate (64%).

#### High frequency (HF)

All data from four studies [51, 52, 54, 56] (92 exercise, 95 control participants) revealed a significant moderate increase (improvement) in HF in favor of the exercise training group (SMD 0.58, 95% CI 0.18, 0.98, *p* = 0.005) (Fig. 3b). The heterogeneity was statistically non-significant (*p* = 0.20), and the inconsistency was moderate (35%).

#### Low frequency-to-high frequency ratio (LF/HF)

All data from three studies [51, 52, 56] (79 exercise, 80 control participants) revealed a significant improvement in LF/HF ratio but the observed improvement was low in favor of the exercise training group (SMD − 0.34, 95% CI − 0.65, − 0.02, *p* = 0.03) (Fig. 3c). The heterogeneity was statistically non-significant (*p* = 0.74) and the inconsistency was low (0%).

#### Heart rate recovery (HRR_1_)

**Fig. 4:**
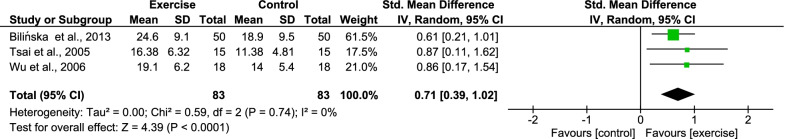
Forest plot for HRR_1_: overall changes in standardized mean difference indices for heart rate recovery in the first minute after exercise (HRR_1_)

All data from three studies [47, 48, 51] (83 exercise, 83 control participants) revealed a moderate significant improvement in HRR_1_ in favor of the exercise training group (SMD 0.71, 95% CI 0.39, 1.02, *p* < 0.0001) (Fig. 4). The heterogeneity was statistically non-significant (*p* = 0.74), and the inconsistency was low (0%).

### Quality of the trials

For all studies, the mean PEDro score was 5.7/10 (good quality). Table 3 provides specifics on how each study scored for each criterion on the quality assessment scale. According to quality ratings, three studies [47, 48, 56] were of good quality, seven [49–55] were of fair quality and one was of excellent quality [56]. Except in two trials [48, 56], most of the studies did not blind participants, therapists or assessors. A method of randomization and concealment of participant group assignment was not provided in 5 studies [49–51, 53, 54]. For reporting between-group differences following an intervention with point estimates and measures of variability all studies received good grades. While just one study [56] used intention-to-treat analysis on dropouts, four studies [51, 52, 55, 56] reported a percentage of dropouts.

**Table 3:** PEDro scores for included trials (*n* = 11)

Trials	Random allocation	Concealed allocation	Groups similar at baseline	Participant blinding	Therapist blinding	Assessor blinding	< 15% dropouts	Intention-to-treat analysis	Between-group difference reported	Point estimate and variability reporter	Total score	Quality
Wu et al. [48]	Yes	Yes	Yes	Yes	No	Yes	Yes	No	Yes	Yes	8/10	Good
Tsai et al. [47]	Yes	Yes	Yes	No	No	No	Yes	No	Yes	Yes	6/10	Good
Legramante et al. [50]	Yes	No	Yes	No	No	No	Yes	No	Yes	Yes	5/10	Fair
Mehani [57]	Yes	Yes	Yes	No	No	No	Yes	No	Yes	Yes	6/10	Good
Bilinska et al. [51]	Yes	No	Yes	No	No	No	No	No	Yes	Yes	4/10	Fair
Ghardashi-Afousi et al. [52]	Yes	Yes	Yes	No	No	No	No	No	Yes	Yes	5/10	Fair
Shao et al. [53]	Yes	No	Yes	No	No	No	Yes	No	Yes	Yes	5/10	Fair
Iellamo et al. [49]	Yes	No	Yes	No	No	No	Yes	No	Yes	Yes	5/10	Fair
Takeyama et al. [54]	Yes	No	Yes	No	No	No	Yes	No	Yes	Yes	5/10	Fair
Mendes et al. [55]	Yes	Yes	Yes	No	No	No	No	No	Yes	Yes	5/10	Fair
Ribeiro et al. [56]	Yes	Yes	Yes	Yes	Yes	Yes	No	Yes	Yes	Yes	9/10	Excellent

## Discussion

### Purpose and main findings

The objective of this systematic review and meta-analysis is to explore the most recent evidence on the influence of exercise training on cardiac autonomic function in post-CABG patients as measured by HRV and HRR. Exercise training improves cardiac autonomic control, according to the majority of the trials. The meta-analysis showed that both HRV and HRR values significantly improved after exercise training.

### HRV

Two frequency domain indices (HF and LF/HF) from our pooled HRV analyses increased significantly indicating improved autonomic function. The HF value increased, indicating a positive change in parasympathetic tone. This elevation denotes a significant change in parasympathetic tone because the parasympathetic nervous system (PNS) is principally responsible for controlling HF [58, 59]. The LF/HF value also underwent a significant improvement (reduction), indicating a better overall balance of sympathetic and parasympathetic function. The lower ratio also suggested a beneficial shift away from sympathetic nervous system (SNS) dominance [60]. LH/HF is still employed widely in research as a marker of sympathovagal balance; however, this finding has been questioned [61]. In the time domain, our meta-analysis revealed a moderately significant rise in SDNN and RMSSD. SDNN shows that HRV has generally improved. An increase in RMSSD is consistent with our finding of increased HF and is not surprising considering their close proximity [59, 62] because it reflects the beat-to-beat fluctuation of heart rate and is connected with parasympathetic tone [58].

The methodological dimensions of HRV evaluation should be considered to clarify at least in part our findings. The health of the ANS is significantly influenced by respiratory and environmental factors [63]. Also, studies conducted in the past showed that HRV indices exhibit circadian variations as well [64]. Therefore, utilizing routine long-term HRV recording values tends to reduce noise and can be a more accurate way to indicate how the PNS functions than using a single-day record [65, 66]. According to Buchheit [63], spectral indices are more sensitive to breathing rhythms and daily fluctuations than time domain indices particularly RMSSD. It becomes imperative to normalize breathing rate when employing frequency domain indices to detect respiratory sinus arrhythmia [67, 68]. None of the studies included in the analysis documented a regulated breathing rate. Most of the studies in our meta-analysis used a single data point to measure HRV. As a result, we discovered that variations in HRV calculation can have a greater impact on the outcomes of investigations using frequency domain indices. Current research suggests that the time domain could be a better way to figure out how well the heart’s ANS is working than the frequency domain. This is because the time domain is less affected by breathing effects and methodological limitations. Additionally, some studies [49–51, 53, 54] lacked concealed allocation, while most of them did not blind participants [47, 49–55, 57], therapist [47–55, 57] or assessors [47, 49–55, 57]. These trials resulted in low-quality scores as blinding is an essential component for the clinical trials. Despite these limitations, the included studies showed positive effect of exercise training for SDNN (*p* = 0.002), RMSSD (*p* = 0.0008), and HF (*p* = 0.005). However, the clinical interpretation based on the present data must be deduced in the light of caution based on the lower bound of the confidence interval obtained for SDNN 0.44 (95% CI: 0.17, 0.71), RMSSD 0.68 (95% CI: 0.28, 1.08) and HF 0.58 (95% CI: 0.18, 0.98).

We did not restrict the exercise duration to investigate the impact of exercise training for a minimal amount of time on cardiac autonomic function. One study compared three different in-patient cardiac rehabilitation programs after surgery. The HRV parameters (SDNN, RMSSD, LF, HF, and LF/HF) changed significantly, and the length of stay in the hospital dropped down [56]. Mendes et al. [55] carried out similar research to determine whether a short-term in-patient exercise program following CABG could enhance cardiac autonomic function. In a study of male CABG patients, Shao et al. [53] looked at how exercise affects the cardiac autonomic function. They found that after 8 weeks of aerobic activity, the time domain of HRV (SDNN, SDANN, RMSSD, and pNN50) was much better in the rehabilitation group than in the control group, which only got standard care. This shows that exercise therapy during rehabilitation will enhance cardiac autonomic nerve regulation following CABG. Additionally, following 2 weeks of residential exercise at 85% of maximum heart rate (HRmax) HRV improved. Baroreflex sensitivity (BRS) and SDANN significantly increased in patients, regardless of whether they had previously experienced a myocardial infarction (MI) [49]. Along with the various benefits of exercise training in the secondary prevention of coronary heart disease, the increased cardiac autonomic function may also be beneficial.

In one study [51], men did aerobic exercise on a cycle ergometer for 6 weeks, 3 days per week, 1 h per session, and at a level that was 70–80% of their HRmax. In the training group, SDNN and HF increased, LF/HF decreased and HRR_1–2_ got improved which showed sympathetic nerve function lowered with parasympathetic dominance. This study depicted that the results are true for a certain group of men who had CABG and a low risk of poor outcomes. Important methodological flaws like allocation and blinding were not clearly stated which affected the quality of the study. In post-CABG men, 6 weeks of HIIT improved cardiac autonomic control, according to a RCT. In comparison with MICT and control, LV-HIIT causes a higher increase in the time domain parameters (SDNN and RMSSD). In addition, HIIT increases HF while reducing LF and LF/HF ratio [52]. However, there is no minimum amount of time needed to get a benefit from exercise training on cardiac autonomic function, as many of the trials of different lengths showed a positive effect on autonomic function. Furthermore, our pooled analysis depicted significant low and moderate increment in LF/HF ratio and HF domain of HRV respectively, indicating enhanced vagal function and sympathovagal balance in favor of the exercise training post-CABG. This pooled finding suggests clinical relevance of exercise in CABG patients irrespective of the length of the exercise training intervention.

### HRR

To meet the metabolic needs of exercise, SNS activity rises when PNS activity declines. After stopping exercise, heart rate drops more and more quickly until it returns back to the resting levels [69, 70]. Most of the improvement in HRR_1_ is due to the reactivation of the PNS, but this fast phase of recovery could also be caused by sympathetic nerves [69]. Parasympathetic reactivation and sympathetic withdrawal are responsible for HRR_2_ enhancements [69]. Improved post-exercise PNS regulation has been shown to protect the cardiovascular system [71] and delayed HRR is a powerful predictor of mortality [72]. The risk of mortality in CAD patients may be reduced as a result of this study [73]. We did not perform any sub-analyses to examine the impact of exercise training on it even though our study showed a statistically significant change in HRR if there was an anomaly at baseline. The value of average baseline HRR_1_ was less than 12 bpm seen in two studies [47, 48], which is usually seen as abnormal following training it shows statistically significant improvements, while one study [51] had a mean baseline HRR_1_ of more than 12 bpm which also depicted significant change post-6 weeks of aerobic training. It is important to highlight that comparisons are made more difficult by the lack of a generally accepted definition of an abnormal HRR or an acceptable recovery treatment. Although HRR_1_ value less than 12 bpm [71, 74] is the most frequently used threshold for abnormality and increased risk, many thresholds have been identified [70].

Since data were reported in an unsuitable form, the results of one study [50] were not pooled for analysis. However, at a descriptive level, the study examined HRR_1_ and HRR_2_ after 2 weeks of aerobic training, and there were insignificant changes in HRR_1_ but significant changes in HRR_2_ indicating enhanced autonomic function. Also, we did not use another study [57] for our meta-analysis because it did not report sufficient data. The author conducted a study to evaluate the impact of in-patient cardiac rehabilitation programs after CABG. Preoperatively, the fourth day following the operation and after the rehabilitation program was completed, HRR_1_ and resting mean six-minute walking distance and predicted peak oxygen consumption were all measured. The results showed improvement in autonomic balance and functional capacity. Furthermore, HRR after exercise depends on a number of factors such as the recovery protocol (active versus passive recovery and recovery posture) [75], the type of exercise [76, 77] and the intensity of the exercise [78]. All of these factors can change the abnormality threshold.

β-blockers may also influence the post-exercise HRR [79], however, to what extent is unknown. In our analysis however, improvements in HRR_1_ were seen in studies [48, 50, 57] in which a limited number of patients were using β-blockers except for one study [51] where majority of the patients were on β-blockers. Combining 12 weeks of exercise training with β-blockers improved HRR in men post-acute MI. This showed that the combination was only more helpful than exercise training alone in the subgroup of patients with a baseline HRR_1_ ≤ 12 bpm [80]. Moreover, HRR_1_ has a strong predictive value in individuals with CAD [81], although HRR_2_ prognostic significance is less clear, despite the possibility that HRR_2_ is superior to HRR_1_ [75]. However, in our analysis only two studies were included that found HRR_2_ was improved after training, indicating that sympathetic activity was lowered while vagal activity improved [69]. Since fewer studies reported HRR_2_ and the diagnostic measures for abnormality are not well defined as for HRR_1_ it is still unclear if the abnormal baseline HRR_2_ would have a better outcome.

Physical activity has many benefits, such as improved breathing and the ratio of ventilation to perfusion, muscle strength, functional capacity and vagal activity [82]. The mechanism of improvement through exercise training in HRV and HRR parameters is unknown. Possible mediators like nitric oxide and angiotensin II which are involved in both the vagal and sympathetic activities of the heart could help exercise training. Nitric oxide has the opposite effect on the vagus nerve than angiotensin II. So, physical activity makes the vagus nerve work more by blocking angiotensin II and making nitric oxide more available [83]. The release of an inhibitory central command or afferent stimulation from baroreflex or chemoreflex functions may be responsible for the considerable decrease in HRR_1_ [84].

### Generalizability

#### Comorbidities

Diabetes is associated with abnormal autonomic function and the incidence of diabetes among patients receiving CABG is 20–23%, according to Russian studies [85, 86] while the US CABG registry estimated the diabetes incidence to be around 46.9% [87]. We did not examine the impact of exercise training differences in diabetic and non-diabetic CABG patients [7, 12, 88–90].

### Gender

Male participants made up the majority of studies, and gender differences in the PNS reactivation during exercise may also exist [91]. Two studies however, found no variations in HRV characteristics depending on gender. Given that there is a clear difference between men and women in the studies and that some people have more than one disease it is important to be careful when applying the results of the review to the whole CABG population.

## Strength and limitation

As far as our knowledge, this is the first systematic review and meta-analysis of CABG patients that totally elucidates the impact of exercise training on cardiac autonomic function. Additionally, the outcomes of this analysis can aid researchers and medical professionals in creating fitness programs for individuals who had CABG. This analysis finds gaps in the current research and suggests that there is a wide range of topics that could be studied about exercise training and the autonomic function of the heart in CABG patients. First of all, HRR and HRV can both be used to predict the outcome, but they both indirectly measures ANS activity, so they cannot reveal the extent of sympathetic or parasympathetic activity. Also, it is still debatable whether HRV tests can give information about how the sympathetic cardiac activity works [61, 92]. Second, three studies [50, 55, 57] were removed from the meta-analysis due to incomplete data reporting, despite the fact that they were of fair quality. Third, not all of the eight studies that made up the meta-analysis provided data on each HRV variable due to substantial heterogeneity. Due to this, the authors only included assessments of cardiac autonomic activity (SDNN, SDANN, RMSSD, LF, HF, and LF/HF) that were often reported (at least twice) in research. Fourth, different ways of evaluating HRV may have affected the analyses and made studies more heterogeneous. Fifth, studies that had been published in languages other than English were also excluded, which could have made publication bias worse. Due to these methodological and assessment limitations the results obtained from the meta-analysis must be extrapolated in the light of caution.

## Conclusions

Exercise training improves HRV and HRR in post-CABG patients indicating improved parasympathetic (vagal) tone and reduced sympathetic response thereby facilitating  cardiac autonomic control and recovery. The findings of this meta-analysis however, should only be utilized to discuss how aerobic exercise impacts male CABG patients autonomic control. Future high-quality trials should include women, emphasize on the methodological elements of HRV and HRR measures and incorporate different forms of exercise training interventions in trials to learn more about how exercise training changes the autonomic function of the heart in CABG patients.

## Data Availability

Data can be available upon reasonable request to the corresponding author.

## Abbreviations

ANS: Autonomic nervous system
BRS: Baroreflex sensitivity
CABG: Coronary artery bypass grafting
CAD: Coronary artery disease
CVD: Cardiovascular disease
HF: High frequency
HRR_1_: Heart rate recovery in the first minute
HRR_2_: Heart rate recovery in the second minute
HRV: Heart rate variability
LF: Low frequency
LF/HF: Low frequency/high frequency
MI: Myocardial infarction
PNS: Parasympathetic nervous system
RMSSD: Root of the mean squared differences between adjacent R-R intervals
SDANN: Standard deviation of the 5 min mean R-R intervals
SDNN: Standard deviation of the R-R intervals
SNS: Sympathetic nervous system
